# Application of 3D scanner to measure physical size and improvement of hip brace manufacturing technology in severe cerebral palsy patients

**DOI:** 10.1038/s41598-023-47665-w

**Published:** 2023-11-24

**Authors:** Jung-Min Kim, Jiwoon Lim, Sun-Young Choi, Sung-Han Rhim, Jaewon Beom, Ju Seok Ryu

**Affiliations:** 1 Department of Health Science and Technology, Graduate School of Convergence Science and Technology, Seoul National University, Seoul, South Korea; 2 Department of Rehabilitation Medicine, Seoul National University Bundang Hospital, 82 Gumi-ro 173 Beon-gil, Bundang-gu, Seongnam-si, Gyeonggi-do 13620 South Korea; 3 Department of Rehabilitation Medicine, Korea University Ansan Hospital, Ansan-si, South Korea; 4 Department of Mechanical Engineering, Dankook University, Yongin, South Korea; 5 Seoul National University College of Medicine, Seoul, South Korea

**Keywords:** Paediatric neurological disorders, Clinical trials, Biomedical engineering

## Abstract

This prospective pilot study aimed to develop a personalized hip brace for treating hip subluxation in children with cerebral palsy. Nineteen children, aged 1–15, with severe cerebral palsy participated in the study. Customized hip braces were created based on 3D scanner measurements and worn for 7 days. The primary outcome, Hip Migration Index (MI), and secondary outcomes, including range of motion (ROM) in the hip and knee joints, pain intensity, satisfaction, discomfort scores, CPCHILD, and wearability test, were assessed. The MI and ROM were assessed at screening and at Visit 1 (when the new hip brace was first worn), while other indicators were evaluated at screening, Visit 1, and Visit 2 (7 days after wearing the new hip brace). The study demonstrated significant improvements in the MI for the right hip, left hip, and both sides. However, there were no statistically significant differences in hip and knee joint ROM. Other indicators showed significant changes between screening, Visit 1, and Visit 2. The study suggests that customized hip braces effectively achieved immediate correction, positively impacting the quality of life and satisfaction in children with cerebral palsy. Furthermore, the hip braces have the potential to enhance compliance and prevent hip subluxation.

**Clinical Trial Registration number:** NCT05388422

## Introduction

Hip joint subluxation in children with cerebral palsy is caused by excessive muscle stiffness around the lower limbs. The main mechanism involves posterior and lateral femoral dislocation^1–4^. This condition typically occurs at around four years of age, with an incidence rate of over 40% before the age of 2 years. This incidence increases as the standing or walking time is delayed or the time spent lying down is increased^1,5,6^. Approximately 63% of GMFCS (Gross Motor Function Classification System Level) IV and V patients with cerebral palsy experience hip subluxation with the risk of subluxation increasing by 3.9% for GMFCS VI and 9.5% for GMFCS V each year^7^. Hence, an X-ray subluxation examination every six months is recommended if cerebral palsy is diagnosed after the age of 2 years^8^. Hip subluxations adversely affect the child’s ability to walk, which is an important factor affecting their future quality of life^7^. Treatments for preventing hip subluxation in children with cerebral palsy vary according to the degree of subluxation^9^. However, the efficacy of these methods remains unclear^10–16^. Therefore, more research is needed to identify effective treatments that can reduce the risk of hip subluxation in this patient population.

Based on previous studies, it has been found that several factors are highly correlated with the development of hip subluxation in children with cerebral palsy. Among these factors are muscle tone, muscle length, and muscle force^17,18^. When the adductor muscles become stiff, the strong contraction of these muscles is expected to increase the risk of hip subluxation by pulling the femoral head out^1,19^. At this time, the joint capsule surrounding the hip joint is stretched and ruptured, resulting in the subluxation of the hip joint^20^. Essentially, the final stage of hip subluxation is the stage in which the muscles and ligaments surrounding the hip joint are stretched^18^.

Considering the results on previous studies, our research team developed the hip brace to protect the hip joint capsule, preventing hip subluxation^21^. However, because patients with severe cerebral palsy have varying body sizes, our team hypothesized that a customized hip brace manufactured using a 3D scanner would improve patient convenience, and intervention effectiveness. Therefore, this study aimed to test this hypothesis by comparing the new 3D-scanned hip brace manufacturing technique with the existing technique.

## Methods

### Study design

This was a single-center, prospective pilot study, conducted from June 2022 to February 2023, at the Seoul National University Bundang Hospital. The study protocol was approved by the Seoul National University Bundang Hospital Institutional Review Board (IRB No.: E-2201-732-001) and registered and approved at clinicaltrials.gov. (ClinicalTrials.gov ID: NCT05388422) All participants and their guardians were enrolled after receiving enough explanation of the research and giving their informed consent. This study was conducted in compliance with the Transparent Reporting of Evaluations with Nonrandomized Designs (TREND) reporting guidelines.

### Participants

The inclusion criteria for participating in the study were as follows: (1) diagnosis of cerebral palsy, (2) age 1–15 years, (3) GMFCS Level IV, V, (4) quadriplegia, or diplegia for more than 6 months, and (5) written consent with permission from the child and caregiver. Patients were excluded if they (1) did not agree to participate in the study, (2) refused the examination, (3) had hip joint surgery experience, (4) were scheduled to undergo surgery during the clinical study, or (5) could not be measured with a 3D scanner.

**Figure 1:**
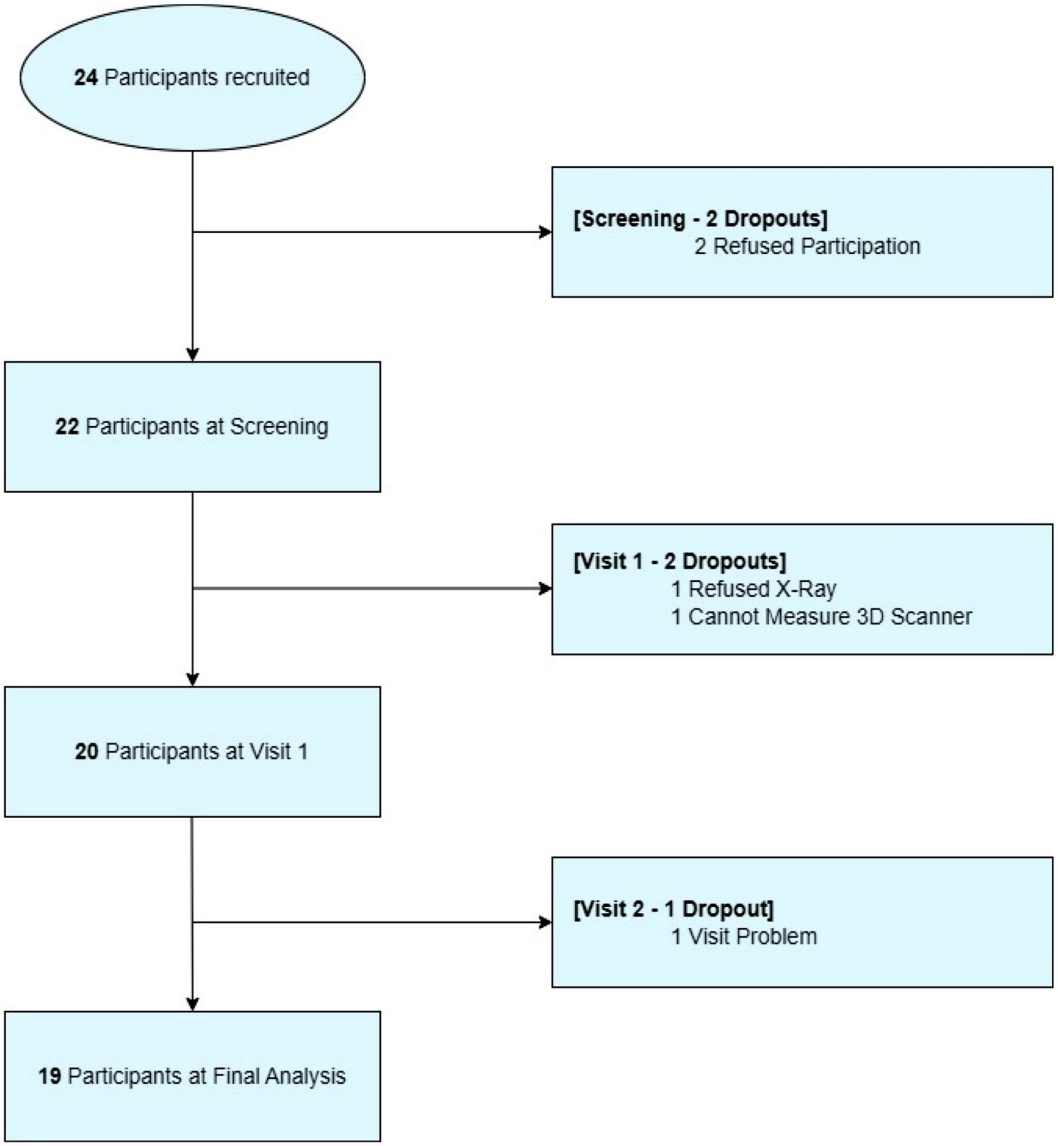
Flowchart of the patients for the study.

A total of 24 patients were initially enrolled in this study, but two patients refused to participate at the screening stage. Two more participants dropped out at the start of wearing the new hip brace period (Visit 1), due to refusal to undergo X-rays (one participant) or inability to be measured by the 3D scanner (one participant). Finally, one participant was unable to attend the last assessment (Visit 2), resulting in a total of 19 patients being included in the study. A detailed flow-chart including the enrollment of the patients is shown in Fig. 1.

### Intervention: hip brace made with 3D Scanner

**Figure 2:**
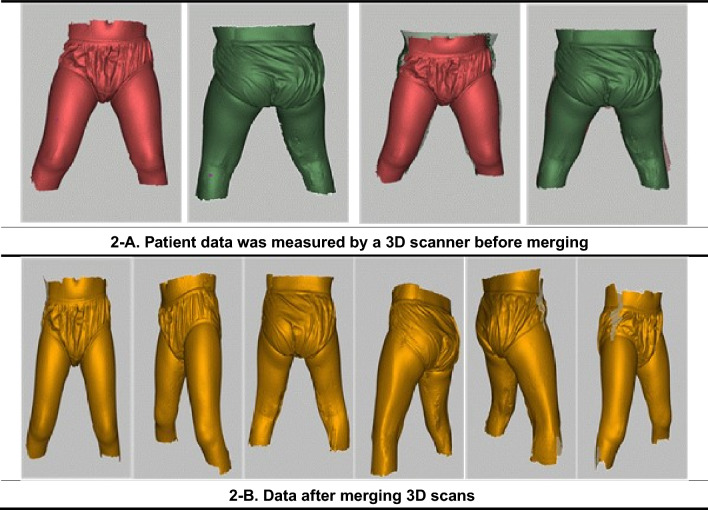
Example of data measured by a 3D scanner and 360-degree merged data.

During the screening process, body size measurements were obtained by means of a 3D scanner. Specifically, the Artect3D-Leo device, manufactured by Artec®, was used due to its ability to perform 3D scanning independently of a computer connection. In this study, it was used to measure the patient’s hip, waist, thigh, and mid-thigh circumference in order to produce a hip brace called RSPROTECTHIP®. Given the challenges faced by children with cerebral palsy in maintaining standing and sitting postures, measurements were taken twice in both the supine and prone positions. In addition, due to the complexities involved in obtaining 360-degree direction measurements data captured from scans performed the 180-degree direction were merged with scans taken as if the patients were standing (360°). When children were unable to comply with the measurement procedures, alternative means were used to confirm data derivation, as shown in Fig. 2. To merge two scanned images of the human body taken at 180-degree intervals, three alignment markers were pre-attached on the patient's body prior to scanning. The alignment markers were then used as reference points to merge the scans using Artec Studio 17 software. Firstly, the raw scan data underwent filtering and smoothing to correct various anomalies, followed by alignment based on the marked reference points. The merged data was then further processed to produce a final image through data cleaning, correction, and pairing.

**Figure 3:**
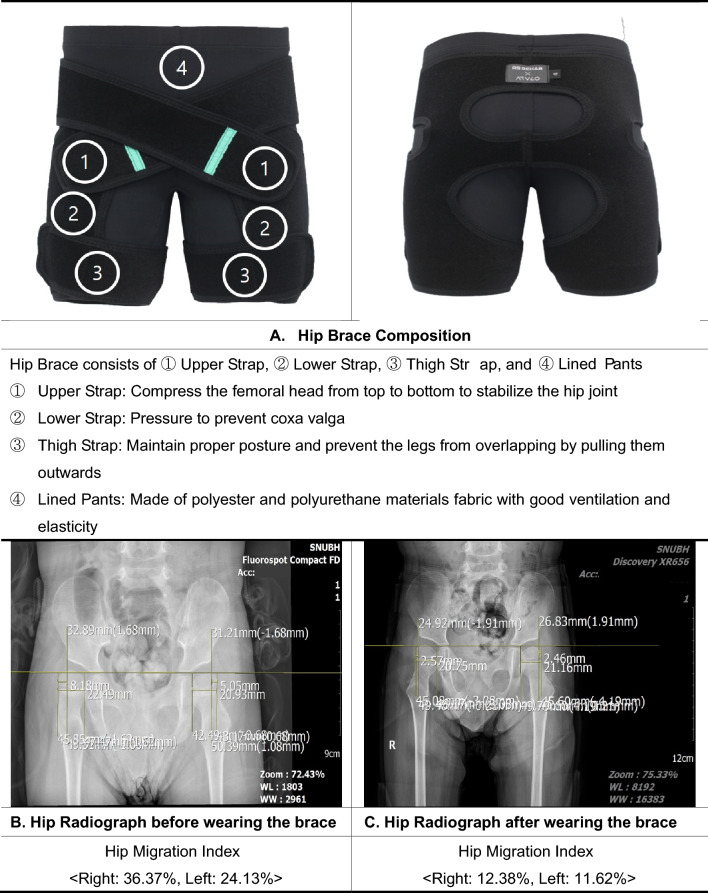
Hip brace composition and example radiographs of a patient.

The hip brace developed by our team has received official approval as a class I medical device from the Ministry of Food and Drug Safety of Korea. (Classification number: B07090.02) The product is composed of an Upper Strap, Lower Strap, Thigh Strap, and Lined Pants made of polyester and polyurethane materials. The Upper Strap compresses the femoral head from top to bottom to stabilize the hip joint, while the Lower Strap is effective in preventing coxa valga. The Thigh Strap acts to maintain proper posture and prevent the legs from overlapping by pulling them outwards. Given the age range of patients who may require this medical device, it is composed of well-ventilated and elastic-lined pants to reduce the patient’s discomfort or any potential displacement when wearing a diaper. A photograph of the hip brace is shown in Fig. 3.

### Outcome variables

The primary outcome, the hip MI was used to measure the ratio of the femoral head not covered by the Acetabulum. The secondary outcomes included the hip and knee joint range of motion (ROM), pain intensity, satisfaction and discomfort score for the hip brace, quality of life (QOL) of the patients and their caregivers, and the wearability test. The hip and knee joint ROM was measured using a goniometer with four indicators: (1) hip abduction with the hip at 90° flexion, (2) hip abduction with the hip at 0° flexion, (3) hip adduction with the hip at 0° flexion, and (4) hip flexion contracture (Thomas test). The pain index was measured on a 10-point scale using the Visual Analog Scale (0: no pain, 10: severe pain). Satisfaction and discomfort scores were measured using the Likert Scale (1: very satisfied or comfort, 5: very dissatisfied or discomfort). The Caregiver Priorities & Child Health Index of Life with Disabilities (CPCHILD) was conducted to measure the QOL of the patients and their caregivers, with higher scores indicating a better QOL. Finally, the Wearability Test consisted of four questions on wearing sensation, five questions about on fit, and four questions on motion suitability evaluation. Each item was scored on 5-point scale, with a total score of 65. The evaluation of the wearing sensation was as follows: (1) convenience of attaching and detaching the Velcro, (2) comfort of the Velcro part, (3) fit of the pants material, and (4) the overall fit. On the other hand, the fit evaluation was as follows: (1) waist, (2) hip, (3) thigh area, (4) pants length, and (5) overall fit. Lastly, the motion suitability evaluation consisted of questions about the suitability of (1) sitting, (2) walking, (3) lying down, and (4) putting on and taking off.

The Hip Migration Index and the Hip and Knee Joint ROM were evaluated on the two different occasions: firstly, during the initial screening (while wearing the existing hip brace), and secondly during Visit 1 (following the introduction of the new hip brace, which was designed with 3D scanner), with guidance provided by a clinician. The other indicators were evaluated three times: at the time of screening, during Visit1 and at Visit2 (which occurred after 7 days of wearing the new hip brace). These other indicators and measurement of the patient’s body size were recorded by two researchers in our team. Face-to-face interviews were conducted during both the screening and Visit 1 phases, which involved the patient visiting the hospital to receive physical measurements and the hip brace. The results of Visit2 were obtained via a phone interview with the patient's guardian.

### Statistical analysis

The baseline characteristics were analyzed using mean ± SD for continuous variables and number (%) for nominal variables. The hip MI and hip and knee joint ROM indicators were compared between the time of screening and Visit 1, using the non-parametric Wilcoxon signed rank test, as the normality assumption was not met based on the Shapiro–Wilk test. The remaining indices also did not meet the normality assumption based on the Shapiro–Wilk test, nor did they meet the assumption of sphericity based on Mauchly’s Test of Sphericity. Therefore, a non-parametric test was conducted. Therefore, the Friedman Test was used to compare the three-time points: screening time, Visit 1, and Visit 2. A post-hoc analysis was then conducted using the Wilcoxon signed-rank test (applying Bonferroni correction: α-level from 0.05 to 0.0167). All statistical significance was stablished at a p-value < 0.05 and a 95% confidence interval. The final results were validated via a reliability test conducted by three evaluators. All data were analyzed using R and R Studio statistical program 4.2.1 version. Statistical analysis of all data were conducted from January to February 2023.

## Results

Table 1 shows the baseline characteristics of all study subjects.

**Table 1:** Demographic and baseline characteristics.

Characteristics	Study (n = 19)
Age (Mean ± SD)	5.73 ± 2.69
1–2 years (N (%))	1 (5.26%)
3–5 years (N (%))	10 (52.63%)
6–15 years (N (%))	8 (42.11%)
Sex (N (%))	
Boys	10 (52.63%)
Girls	9 (47.37%)
GMFCS level (N (%))	
Level IV	6 (31.58%)
Level V	13 (68.42%)
Surgery experience except hip joint surgery (N (%))	
Yes	1^a^ (5.26%)
No	18 (94.74%)
Experience with hip brace (N (%))	
Yes	10 (52.63%)
No	9 (47.37%)

^a^A patient who has previously had internal rotator cuff surgery.

**Figure 4:**
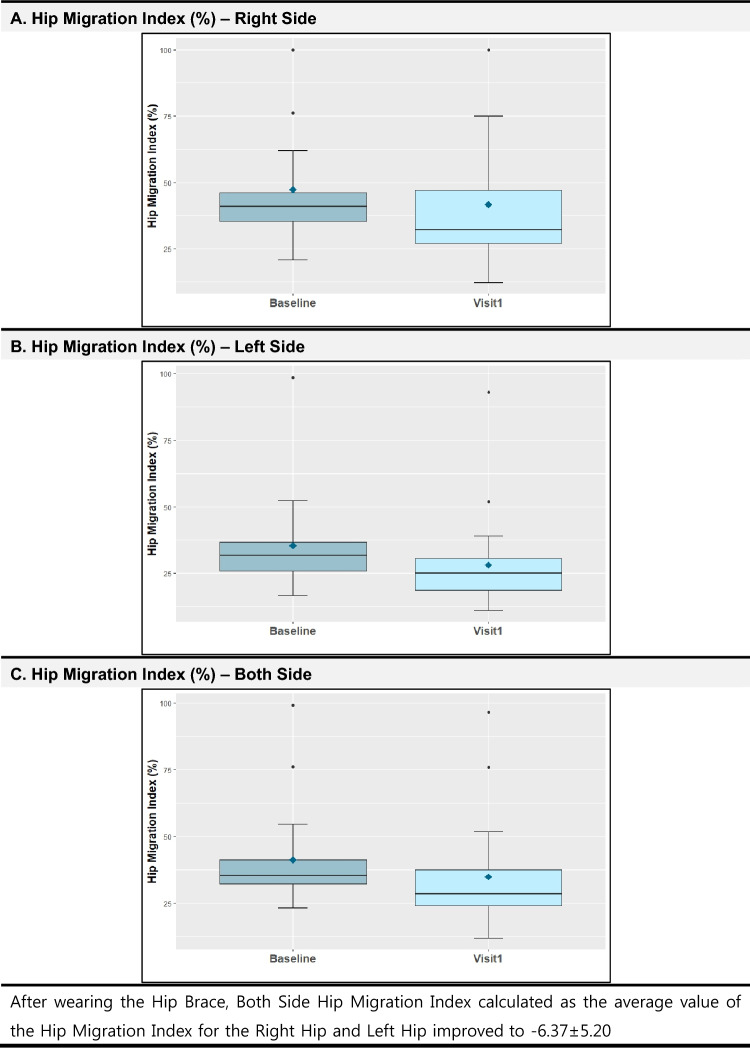
Hip migration index at baseline and wearing the hip brace.

Table 2 and Fig. 4 shows the primary outcomes, consisting of the change in the hip MI. Regarding this outcome, the change of right hip MI between the screening and Visit 1 was − 5.53 ± 8.70 (p-value: 0.004), while the left hip MI changed to − 7.21 ± 6.86 (p-value < 0.001), indicating statistical significance in both cases. The both side hip MI calculated as the average value for the right hip and left hip also improved to − 6.37 ± 5.20 (p-value < 0.001). The evaluation the hip and knee joint ROM as a secondary outcome revealed no significant changes.

**Table 2:** Hip migration index and ROM changes before and after wearing the hip brace.

	Mean ± SD				Estimation of difference (95% CI)
	Before^a^ (Screening)	After^b^ (Visit 1)	Difference	p-value	
Hip migration index (%)					
Right	47.23 ± 22.37	41.70 ± 25.44	− 5.53 ± 8.70	0.004*	− 6.39 (− 10.86, − 1.45)
Left	35.42 ± 17.57	28.21 ± 18.62	− 7.21 ± 6.86	p < 0.001*	− 7.11 (− 10.47, − 3.66)
Both	41.33 ± 18.56	34.95 ± 20.82	− 6.37 ± 5.20	p < 0.001*	− 6.16 (− 8.87, − 3.56)
Hip and Knee Joint ROM (Range of motion of the hip and knee joint)					
Hip abduction with hip at 90° flexion	53.16 ± 14.26	54.74 ± 7.35	1.58 ± 8.00	0.605	2.50 (− 7.50, 20.00)
Hip abduction with hip at 0° flexion	41.05 ± 6.99	41.05 ± 6.58	0.00 ± 3.73	1.000	− 0.00 (− 2.50, 5.00)
Hip adduction with hip at 0° flexion	25.00 ± 0.00	25.00 ± 0.00	0.00 ± 0.00	NA	NaN (NaN, NaN)
Hip flexion Contracture (Thomas test)	132.63 ± 10.32	134.21 ± 3.44	1.58 ± 6.88	1.000	30.00^c^ (NaN, NaN)

*Hip Migration Index (%) showed significant changes on the Right, Left, and Both Sides.

^a^Measured without wearing a hip brace

^b^Measured wearing a hip brace

^c^Only one sample has a difference: 30.00.

Table 3 presents the results of pain intensity, hip brace satisfaction, discomfort, CPCHILD, and wearability measurements. As a result of comparing screening, Visit1, and Visit2 phases, a significant effect was found in pain intensity (p-value: 0.001, χ^2^ = 13.43, df = 2), hip brace satisfaction (p-value: 0.001, χ^2^ = 13.61, df = 2), discomfort (p-value 0.001, χ^2^ = 13.56, df = 2), CPCHILD (p-value: 0.002, χ^2^ = 12.25, df = 2), and wearability test (p-value: 0.014, χ^2^ = 8.60, df = 2). Particularly, the result of post-hoc analysis by dividing the three-time points into two-time points (Bonferroni correction: α-level from 0.05 to 0.0167) for pain intensity, hip brace discomfort, and CPCHILD showed significant improvement at the Visit 2 when compared to the screening phase.

**Table 3:** Result of questionnaire evaluation.

	Mean ± SD	Estimation of difference (95% CI)	p-value	
			Between two periods^†^	Between three periods
Pain intensity^a^				
Visit1–Screening	− 1.79 ± 2.68	− 2.50 (− 4.00, − 0.50)	0.0146**	0.001* (χ^2^ = 13.43, df = 2)
Visit2–Visit1	− 0.79 ± 1.27	− 1.00 (− 2.00, − 1.00)	0.0186	
Visit2–Screening	− 2.58 ± 2.52	− 3.00 (− 4.50, − 1.50)	0.0014**	
Hip brace satisfaction^b^				
Visit1–Screening	− 0.40 ± 1.07	− 0.50 (− 2.00, 1.00)	0.2795	0.001* (χ^2^ = 13.61, df = 2)
Visit2–Visit1	− 0.17 ± 0.79	− 1.00 (− 1.00, 0.00)	0.4070	
Visit2–Screening	− 0.80 ± 0.92	− 1.50 (− 2.00, − 1.50)	0.0533	
Hip brace discomfort^b^				
Visit1–Screening	− 0.90 ± 0.57	− 1.00 (− 1.00, − 1.00)	0.0083**	0.001* (χ^2^ = 13.56, df = 2)
Visit2–Visit1	− 0.06 ± 1.11	0.00 (− 1.00, 1.00)	0.7055	
Visit2–Screening	− 1.50 ± 1.08	− 2.00 (− 2.50, − 1.00)	0.0132**	
CPCHILD				
Visit1–Screening	5.08 ± 11.27	3.68 (− 0.98, 9.56)	0.1365	0.002* (χ^2^ = 12.25, df = 2)
Visit2–Visit1	4.80 ± 7.52	5.39 (1.72, 8.33)	0.0090**	
Visit2–Screening	9.88 ± 14.23	9.55 (3.68, 16.42)	0.0021**	
Wearability test^c^				
Visit1–Screening	7.40 ± 9.02	7.00 (1.00, 14.50)	0.0371	0.014* (χ^2^ = 8.60, df = 2)
Visit2–Visit1	4.95 ± 13.66	6.00 (− 0.50, 11.50)	0.0533	
Visit2–Screening	10.70 ± 14.15	13.50 (− 4.00, 19.00)	0.0829	

^a^Pain Intensity: from 0 (no pain) to 10 (severe pain)

^b^Satisfaction and Discomfort: from 1 (very satisfied or comfort) to 5 (very dissatisfied or discomfort)

^c^Wearability Test: consisted of 4 questions on wearing sensation, 5 questions about fit, and 4 questions on motion suitability evaluation. Each item was scored on a 5-point scale, with a total score of 65. The responses for each item are as follows: from 1 (very discomfort or fits very poorly) to 5 (very comfort or fits very well).

^†^p-value (Between Two Periods): a post-hoc analysis was conducted using Wilcoxon Signed Rank Test. (Bonferroni correction: α-level from 0.05 to 0.0167)

*There were significant effects in all indicators (pain intensity, hip brace satisfaction, discomfort, CPCHILD, and wearability test)

**These indicators showed significant improvements after the post-hoc analysis.

## Discussion

This study demonstrated that a patient-customized hip brace using a 3D scanner had a significant effect on improving hip subluxation compared with baseline. The primary outcome of the study, the hip MI, showed significant improvement on both the right and left sides, as well as bilaterally. This improvement not only affected the subluxation of the patient’s hip joint, but also their QOL. Compared to existing hip braces, this study proves that the customized hip brace shows significant effects on all aspects of the patients’ and their caregivers’ QOL. The commercialization of this medical device is expected to provide a breakthrough in treatment paradigms.

Previous studies have investigated interventions for hip subluxation in children with cerebral palsy have been studied from three perspectives: developmental, subluxation-inducing, and protective factors^13^. Developmental factors include the weight applied to the hip joint^22–26^, state of motor function (GMFCS level)^5,26^, and balance of forces applied to the hip joint.^19^ Weight-bearing exercises in children with developmental disabilities, such as cerebral palsy, have shown to promote acetabular development, demonstrating an intervention effect through exercise programs or postural correction methods^22–24^. Additionally, several studies have reported that the risk of hip subluxation increases as the GMFCS levels increase. In particular, in children with GMFCS V, the risk of subluxation was approximately 2.5 to 3 times higher than that in children with GMFCS III-IV^5,26,27^. As for the balance of hip migration and forces applied to the hip joint, there is a paper reporting that hip flexor and adductor muscle elasticity are correlated with the occurrence of hip migration^19^. Depending on the balance of the forces applied to the hip joint (abductors, extensors, and adductor muscles), coxa valga, femoral anteversion, and acetabular dysplasia may occur^28–33^.

There are several protective factors that can help prevent hip subluxation, including reducing laxity of the hip ligaments and preventing muscle weakness around the hip joint^25,34^. In fact, there have been efforts to develop and utilize a hip brace as a preventive measure for children with cerebral palsy^14,35,36^. However, despite various perspective and attempts, the level of evidence for the effectiveness of these interventions is still not yet high. This is based on the results of a meta-analysis, which also revealed a lack of clear intervention strategies for hip subluxation in children with cerebral palsy^15^.

In a previous study, we developed a hip brace that could prevent the progression of hip subluxation and reinforce the protection of the capsule and ligaments. Although previous studies using existing hip braces, such as the SWASH did not show a significant effect on hip subluxation, our hip brace (RSPROTECTHIP®), which was developed based on the mechanism of hip subluxation, showed a statistically significant effect^14,37^. This was confirmed through a multi-center clinical study, where it was proven that our hip brace had a preventive effect on hip subluxation. In the control group not wearing the hip brace, subluxation progressed by 5.9 ± 7.4% at 6 months of follow-up and 9.4 ± 10.9% at 1 year. In contrast, in the experimental group wearing the hip brace, the results showed that the subluxation decreased by − 2.7 ± 6.9% at 6 months of follow-up and by − 3.3 ± 6.9% at 1 year^21^. However, the previous study had a limitation in that it was mediated according to the typical brace size for children by age.

Children with cerebral palsy, unlike healthy children, have varied body sizes due to pathophysiological mechanisms, making it challenging to create a hip brace that fits their body^38^. To address this issue, this pilot study was conducted in order to perform individual patient body measurements using a 3D scanner. Using a 3D scanner for body measurements offers advantages such as comfortable fitting, the ability to simulate mechanical properties and pressure, and it often achieves comparable accuracy to manual measurements by human experts. Additionally, 3D scanning is non-contact, ensuring individual privacy protection^39–47^. The purpose of this study was to create a customized hip brace, taking the advantages of the 3D scanner, which was found to be superior to the standardized sizes appropriate for the age of the patient used in previous studies. The customized hip brace showed superiority in immediate correction, QOL, and satisfaction^21^. Further studies will be conducted to prove the significant effects of this custom-made hip brace on hip subluxation, delay in the surgical period, and the improvement of the patient’s QOL through long-term intervention. Additionally, comparison of the intervention effect between the existing prototype and this custom-made hip brace through a 3D scanner will be made.

### Limitations

This study had some limitations. First, the 3D scanning process is affected by the movement of the children with cerebral palsy, which resulted in inaccurate groin scans. We attempted to compensate for this by using the fix holes function, but the accuracy of the result was not satisfactory due to the large and wide area of the missing data. Moreover, the panty line of the patient showed an abnormal height value owing to an error between the anterior and posterior data. Second, due to the nature of the disease, the patient’s posture is often unstable, and the angle or position of the legs and joints may change when lying on the ceiling (supine) or on the stomach (prone). This led to data errors and an increased error rate during merging. Third, there was an error rate between the data measured using the 3D scanner and the body measurement data. Although the previously planned 3D scanning pattern range within 10%, the limitation of the 3D scanning system increased the accuracy, to approximately 15% accuracy (Supplement Table 2). Discrepancies in body sizes were measured and recorded, comparing 3D scanning data with marked reference points to direct body measurements. These discrepancies were largely due to challenges in controlling patient movements during scanning and unclear merging reference points. Following a statistical analysis of data from participants, we determined an acceptable error margin of around 15%. Since this was an immediate, short-term follow-up study, further research is needed to confirm whether this custom-made hip brace will have better long-term effect than the existing standardized hip braces.

## Conclusions

In summary, the results of this pilot study suggest that a customized hip brace created using a 3D scanner is effective for immediate correction of hip subluxation in children with cerebral palsy. This medical device showed excellent results for various indicators such as patient QOL and satisfaction. The used of a customized brace is also expected to improve patient compliance by reducing discomfort. Therefore, this study suggests that a custom-made hip brace could be a more effective intervention for preventing hip subluxation in children with cerebral palsy in the future.

### Supplementary Information

Supplementary Table 1.

Supplementary Table 2.

## Data Availability

All data in this study is available after de-identification upon request. The data that support the findings of this study are available from the first author, Jung-Min Kim (owljm@snu.ac.kr), upon reasonable request.
