# Supplementary material for: Application of 3D scanner to measure physical size and improvement of hip brace manufacturing technology in severe cerebral palsy patients
Source: Sci Rep. 2023 Nov 24;13:20691. doi: 10.1038/s41598-023-47665-w (PMC10673940; doi:10.1038/s41598-023-47665-w)
Supplement: Supplementary file 1 — Supplementary Table 1. [file 41598_2023_47665_MOESM1_ESM.docx]

[Supplement Table 1. Hip migration difference analysis depending on GMFCS level]

|  | **Within Group (Mean±SD)** | | | | **Between Group (Mean±SD)** | | | | | |
| --- | --- | --- | --- | --- | --- | --- | --- | --- | --- | --- |
|  | **Before**  **(Screening)** | **After**  **(Visit 1)** | **After – Before** | **p-value** | **Before (Screening)** | | **After (Visit 1)** | | **After - Before** | |
|  |  |  |  |  | **Estimation of Difference (95% CI)** | **p-value** | **Estimation of Difference (95% CI)** | **p-value** | **Estimation of Difference (95% CI)** | **p-value** |
| **Hip Migration Index – Right (%)** | | | | | | | | | | |
| GMFCS Level 4 | 36.07±8.24 | 29.50±5.30 | -6.57±4.71 | 0.03* | 8.74  (-3.67, 37.50) | 0.15 | 11.54  (-5.18, 42.45) | 0.40 | 1.88  (-8.12, 9.74) | 0.51 |
| GMFCS Level 5 | 52.38±25.12 | 47.33±29.16 | -5.05±10.18 | 0.04* |  |  |  |  |  |  |
| **Hip Migration Index – Left (%)** | | | | | | | | | | |
| GMFCS Level 4 | 31.60±5.39 | 20.35±4.66 | -11.25±8.00 | 0.03* | 0.06  (-7.61, 13.08) | 1.00 | 7.06  (-3.75, 18.14) | 0.21 | 5.21  (-1.45, 12.80) | 0.18 |
| GMFCS Level 5 | 37.19±20.98 | 31.83±21.58 | -5.35±5.66 | 0.01* |  |  |  |  |  |  |
| **Hip Migration Index – Both (%)** | | | | | | | | | | |
| GMFCS Level 4 | 33.83±3.11 | 24.92±4.62 | -8.91±3.60 | 0.03* | 3.29  (-3.52, 20.70) | 0.24 | 7.68  (-3.02, 25.46) | 0.09 | 4.80  (-0.84, 8.73) | 0.09 |
| GMFCS Level 5 | 44.78±21.72 | 39.58±23.83 | -5.20±5.65 | 0.002* |  |  |  |  |  |  |
| * Within each GMFCS level, there were significant differences before and after (p-value < 0.05). However, there were no significant differences observed between different GMFCS levels. | | | | | | | | | | |
