# Supplementary material for: Application of 3D scanner to measure physical size and improvement of hip brace manufacturing technology in severe cerebral palsy patients
Source: Sci Rep. 2023 Nov 24;13:20691. doi: 10.1038/s41598-023-47665-w (PMC10673940; doi:10.1038/s41598-023-47665-w)
Supplement: Supplementary file 2 — Supplementary Table 2. [file 41598_2023_47665_MOESM2_ESM.docx]

[Supplement Table 2. Difference between 3D Scanner Measurement Result and Hip Brace Size]

|  | **Mean Difference**  **(3D Scanner Measurement Result - Hip Brace Size)** | **Mean Error Rate (%)**  **(Mean Difference / 3D Scanner Measurement Result)** |
| --- | --- | --- |
| Hip Circumference | 1.89±3.93 | 3.45±7.74 |
| Waist Circumference | 8.29±3.43 | 17.69±7.08 |
| Thigh Circumference | 1.92±4.10 | 4.96±13.36 |
| Mid-Thigh Circumference | -0.39±3.41 | -3.87±14.88 |
